# Pre-hospital plasma in haemorrhagic shock management: current opinion and meta-analysis of randomized trials

**DOI:** 10.1186/s13017-019-0226-5

**Published:** 2019-02-15

**Authors:** Federico Coccolini, Giacinto Pizzilli, Davide Corbella, Massimo Sartelli, Vanni Agnoletti, Vanessa Agostini, Gian Luca Baiocchi, Luca Ansaloni, Fausto Catena

**Affiliations:** 1 General, Emergency and Trauma Surgery, Bufalini Hospital, Viale Ghirotti 268, 47521 Cesena, Italy; 2 ICU department, Bufalini Hospital, Cesena, Italy; 3 ICU department, Papa Giovanni XXIII Hospital, Bergamo, Italy; 4 General Surgery department, Macerata Hospital, Macerata, Italy; 5 Transfusional and Immunohaematological disorders department, Bufalini hospital, Cesena, Italy; 6 Department of Clinical and Experimental Sciences, University of Brescia, Brescia, Italy; 7 Emergency surgery department, Parma University Hospital, Parma, Italy

**Keywords:** Haemorrhagic, Shock, Pre-hospital, Treatment, Meta-analysis, Trauma, Management

## Abstract

**Background:**

Trauma-induced coagulopathy is one of the most difficult issues to manage in severely injured patients. The plasma efficacy in treating haemorrhagic-shocked patients is well known. The debated issue is the timing at which it should be administered. Few evidences exist regarding the effects on mortality consequent to the use of plasma alone given in pre-hospital setting. Recently, two randomized trials reported interesting and discordant results. The present paper aims to analyse data from those two randomized trials in order to obtain more univocal results.

**Methods:**

A systematic review with meta-analysis of randomized controlled trials (RCTs) of pre-hospital plasma vs. usual care in patients with haemorrhagic shock.

**Results:**

Two high-quality RCTs have been included with 626 patients (295 in plasma and 331 in usual care arm). Twenty-four-hour mortality seems to be reduced in pre-hospital plasma group (RR = 0.69; 95% CI = 0.48–0.99). Pre-hospital plasma has no significant effect on 1-month mortality (RR = 0.86; 95% CI = 0.68–1.11) as on acute lung injury and on multi-organ failure rates (OR = 1.03; 95% CI = 0.71–1.50, and OR = 1.30; 95% CI = 0.92–1.86, respectively).

**Conclusions:**

Pre-hospital plasma infusion seems to reduce 24-h mortality in haemorrhagic shock patients. It does not seem to influence 1-month mortality, acute lung injury and multi-organ failure rates.

**Level of evidence:** Level I

**Study type:** Systematic review with Meta-analysis

## Background

Trauma-induced coagulopathy is one of the most difficult issues to deal with in severely injured patients. It leads to uncontrolled bleeding and/or uncontrollable fibrinolysis due to depletion in pro- and anti-coagulant factors. The efficacy of plasma in the management of patients suffering from haemorrhagic shock is well known, but timing of its administration is less clear. Several studies clarify plasma and blood product beneficial effect in restoring a better coagulation capacity in haemorrhagic and severely deranged patients [1–7]. Few evidences exist regarding the effects of the use of plasma alone in pre-hospital setting [8]. Recently, two randomized trials reported interesting and discordant results [9, 10]. The present paper aims to summarize data from those two studies in order to obtain more univocal results.

## Methods

### Literature search strategy

Electronic searches were performed using MEDLINE, Embase (1988–August 2018), PubMed (January 1980–August 2018), Cochrane Central Register of Controlled Trials (CCTR), Cochrane Database of Systematic Reviews (CDSR) and CINAHL from (1966–2018). The search terms were ‘hemorrhagic shock’, ‘pre-hospital’, ‘plasma’, ‘management’, ‘protocol’, ‘standardized’, ‘randomized trial’, and ‘meta-analysis’, combined with AND/OR. Research included also all the MeshTerms. No search restrictions were imposed. The reference lists of all retrieved articles were reviewed for further identification of potentially relevant studies. Review articles were also obtained to determine other possible studies. Duplicate published trials with accumulating numbers of patients or increased lengths of follow-up were considered only in the last or at least in the more complete version.

### Selection criteria

We included in the systematic review and meta-analysis studies where patients with early haemorrhagic shock were randomly assigned to receive either plasma in pre-hospital setting or standard care. No language restrictions have been applied. Eligibility for study inclusion and study quality assessment were performed independently by two authors (FeCo, GP). Study data were extracted onto standard forms independently by two authors (FeCo, GP). Discrepancies between the two investigators were resolved by discussion and evaluation of the question with a senior investigator. The final results were reviewed by three other investigators (FCa, VAgn, VAg).

The primary outcome measures for the meta-analysis were mortality at 24 h and at 1 month. Secondary outcome was morbidity (acute lung injury and multi-organ failure). The two studies reported 30 days mortality [10] and 28 days mortality [9]; for the purpose of the study, it was judged comparable so the two time points were considered together in the pooled analysis.

### Assessment of risk of bias

There is a potential risk of overestimating the beneficial treatment effects of randomized controlled trial (RCT) with a resultant risk of bias. The risk of bias was assessed comprehensively according to guidelines of the Cochrane Collaboration [11], and six items have been considered relevant (Table 1): (1) whether the method of allocation was truly random, (2) whether there was proper allocation concealment, (3) whether the groups were similar at baseline, (4) whether the eligibility criteria were documented, (5) whether loss to follow-up in each treatment arm was specified and (6) whether intention-to-treat analysis was conducted. We ranked the studies according to the number of positive answers. A high-quality study must have six positive answers, a fair quality one five or four, and a low quality less than four.

**Table 1:** Study quality

Study (ref.), year	Randomisation	Allocation concealment	Homogeneous baseline characteristic	Eligibility criteria	Loss to follow-up and drop-out described	Intention-to-treat analysis	Study quality
Moore [9], 2018	Yes	Yes	Yes	Yes	Yes	Yes	High
Sperry [10], 2018	Yes	Yes	Yes	Yes	Yes	Yes	High

### Statistical analysis

Data from the individual eligible studies were entered into a spreadsheet for further analysis. Review Manager (RevMan) (version 5.1. Copenhagen: The Nordic Cochrane Centre, The Cochrane Collaboration, 2011) was used to perform the statistical analysis. Pooled odds ratio (OR) or risk ratio (RR) were calculated for discrete variables, and weighted mean difference was used for continuous variables. The fixed-effects and random-effects models were used to calculate the outcomes [12, 13]. In case of significant statistical heterogeneity, only the results of the random-effects model were reported. Heterogeneity amongst the trials was determined by means of the Cochrane Q value and quantified using the *I*^2^ inconsistency test.

#### Trial sequential analysis

Random error is a source of misleading results in meta-analysis especially when there is accumulating evidence from multiple trials, and therefore, multiple tests are performed. To overcome this problem, we opted to perform a trial sequential analysis. This methodology combines a new sample size calculation and sequential monitoring boundaries. The sample size calculation is aimed to include an information size at least as large as the sample size of an adequately powered single trial to reduce the risk of random error and is updated when a new trial is added to the meta-analysis in order to take into account for heterogeneity of the studies and number of events. We considered an alpha of .05 and a power of .80 to have a reduction from .25 to .15 of mortality at 24 h and 30 days. The sequential monitoring boundaries take into account the possible effect of multiple comparisons when trials are added to a meta-analysis. These boundaries can be used in cumulative meta-analyses to distinguish real effects from random errors. Boundaries were calculated according to O’Brien – Fleming alpha-spending function and futility one according to O’Brien – Fleming beta-spending function. Trial sequential analysis was performed using Trial Sequential Analysis Viewer (TSA Viewer) [Computer program] version 0.9.5.10 Beta. Copenhagen: Copenhagen Trial Unit, Centre for Clinical Intervention Research, Rigshospitalet, 2016.

## Results

**Fig. 1:**
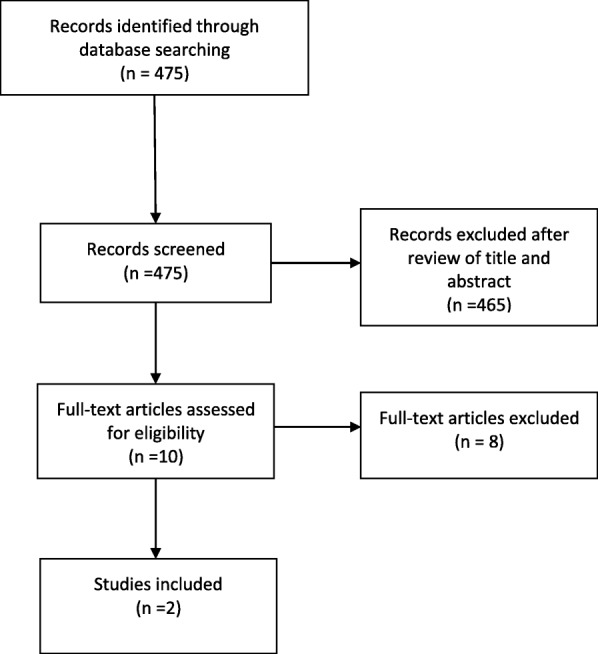
PRISMA flow diagram

Results from the literature systematic search and analysis are shown in Fig. 1.

Two RCTs fulfilled the inclusion criteria and were included in the meta-analysis (publication date 2018) [9, 10]. There were a total of 626 patients (295 randomized to receive plasma and 331 randomized to receive standard care).

### Patient management

In both studies, inclusion criteria were similar and the eligible patients were severely injured adults (age > 18 and < 90 years), with systolic blood pressure (SBP) 70 mmHg or lower or 71–90 mmHg and hearth rate 108 beats per min thought to be due to acute blood loss, either before the arrival of air medical transport or any time before arrival at the trauma centre. The exclusion criteria were prisoner status, known pregnancy, isolated gunshot to the head, asystole or cardiopulmonary resuscitation cardiac arrest that lasted longer than 5 min before randomisation, impossibility to establish an intravenous or intra-osseous access, documented cervical cord injury, burns over more than 20% of total body surface area, if they were being admitted as an inpatient at an outside referral hospital, known objection to blood products, opt-out bracelets or necklaces or family objection to the patient’s enrolment.

In both studies, patients were randomized to plasma-based resuscitation (2 U of FFP ~ 250 mL each) or standard resuscitation protocol according to the local rules.

### Quality of trials

There was good agreement between the reviewers (FeCo and GP) about the eligibility and quality of the studies. Table 1 demonstrates the quality of the two included RCTs [9, 10].

Both RCTs [9, 10] were ranked as high-quality studies (Table 1). The method of allocation concealment was adequate; randomisation was performed on a central site and transmitted to treatment providers by telephone or sealed opaque envelopes, and the baseline features were similar between treatment groups thus reducing the clinical heterogeneity. All RCTs specified the eligibility criteria for patients to be enrolled and the number of patients lost to follow-up in each treatment group and analysed the data on an intention-to-treat basis, whereby participants were analysed in the groups to which they were initially randomized and the attrition bias risk was low. Blinding after allocation was impossible because of the nature of the trials. Significant publication biases were not recognized.

### Mortality at 24 h

**Fig. 2:**
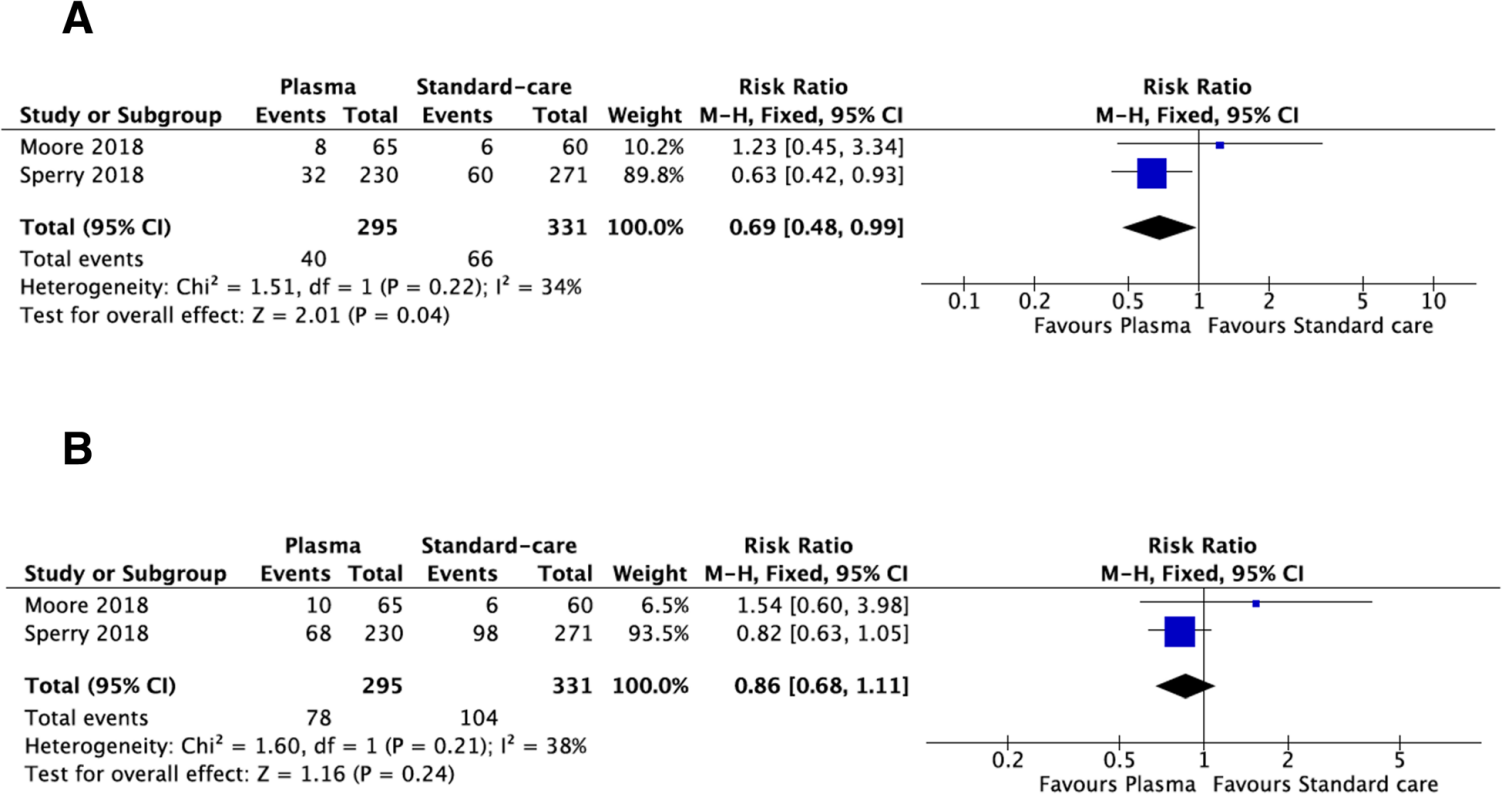
Mortality outcomes: 24-h mortality (**a**), 1-month mortality (**b**)

Two hundred ninety-five patients received pre-hospital plasma and 331 usual care (Fig. 2). There was no statistical heterogeneity between studies. In the fixed-effects model, the 24-h mortality was significantly higher in the standard treatment group (RR = 0.69; 95% CI = 0.48–0.99).

### Mortality at 1 month

Two hundred ninety-five patients received pre-hospital plasma and 331 usual care (Fig. 2). There was no statistical heterogeneity between studies. In the fixed-effects model, the 1-month mortality was not significantly different between the two arms (RR = 0.86; 95% CI = 0.68–1.11).

### Acute lung injury

**Fig. 3:**
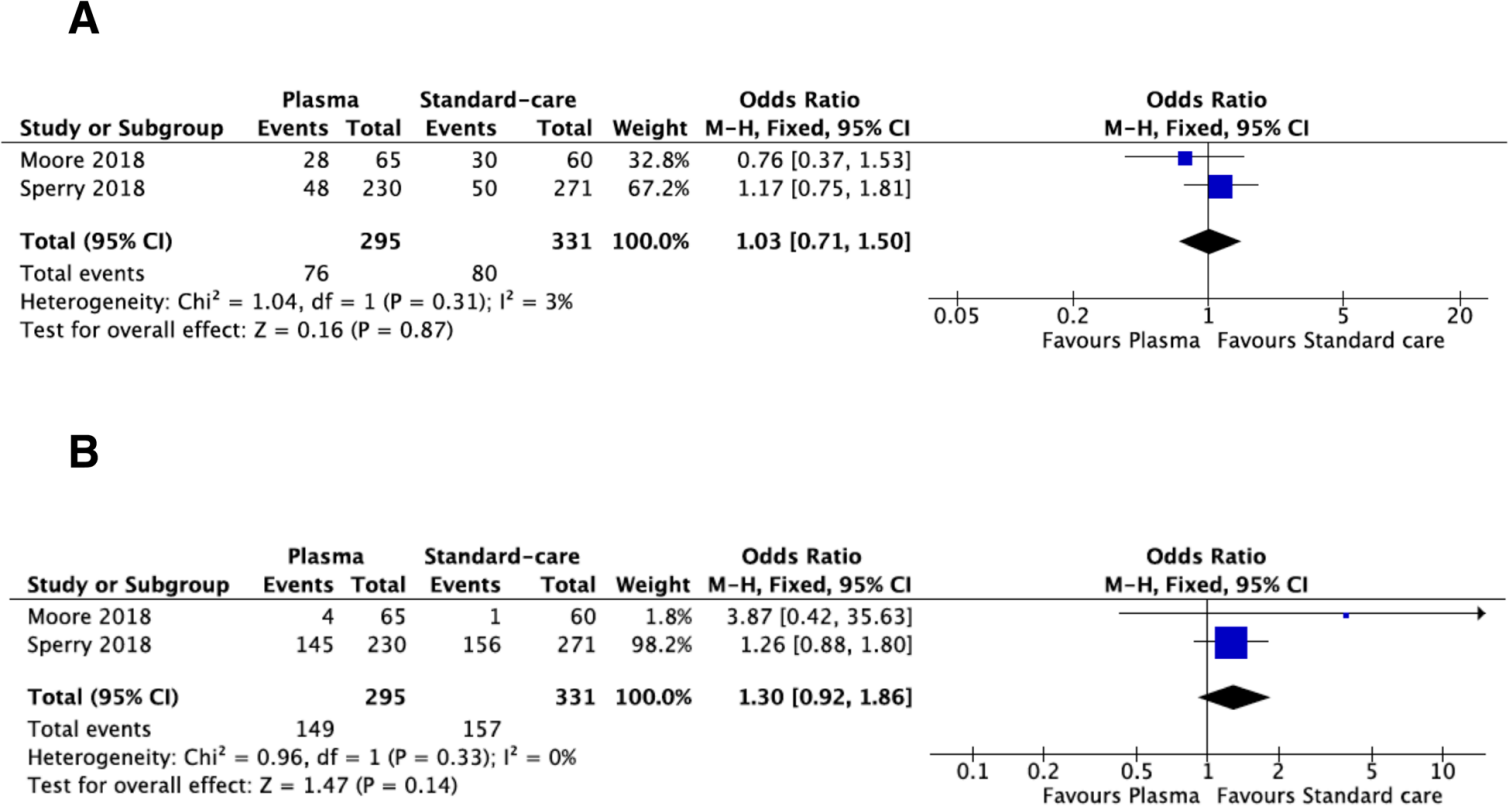
Morbidity outcomes: acute lung injury (**a**), multi-organ failure (**b**)

Two hundred ninety-five patients received pre-hospital plasma and 331 usual care (Fig. 3). There was no statistical heterogeneity between studies. In the fixed-effects model, the acute lung injury rate was not significantly different between the two arms (OR = 1.03; 95% CI = 0.71–1.50).

### Multi-organ failure

Two hundred ninety-five patients received pre-hospital plasma and 331 usual care (Fig. 3). There was no statistical heterogeneity between studies. In the fixed-effects model, the multi-organ failure rate was not significantly different between the two arms (OR = 1.30; 95% CI = 0.92–1.86).

### Trial sequential analysis

**Fig. 4:**
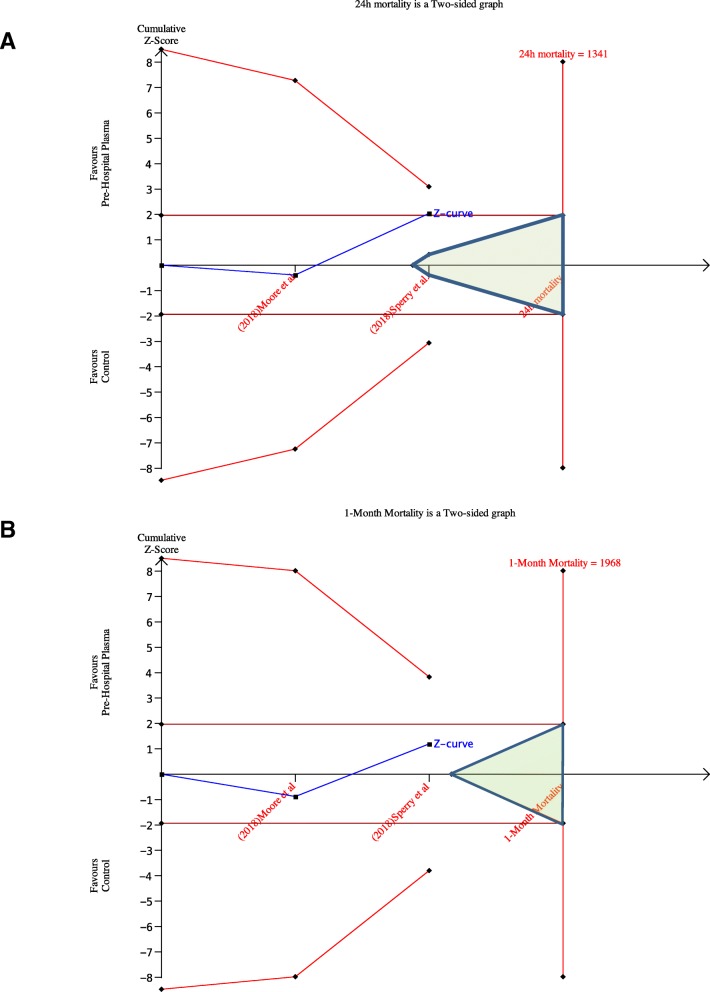
Trial sequential analysis: 24-h mortality (**a**), 1-month mortality (**b**). [How to read the figure: In blue is the cumulative *Z*-curves calculated after including subsequent trial into the meta-analysis. A reference line at two-sided *Z* = 1.96 (equal to a *p* = .05) is drawn, and it is usually considered the significant result threshold in meta-analysis. Trial sequential monitoring boundaries are reported in red. To obtain reliable evidence (pros or cons of the intervention), the cumulative *Z*-score must cross the red line. The futility area is dyed in green. If the cumulative *Z*-score hit the futility area, the result of the meta-analysis is for no effect (neither negative nor positive) of the intervention. On the far right of the figure is the line with the sample size calculation according to the trial sequence analysis methodology]

Neither 24-h mortality nor 1-month mortality crossed the sequential monitoring boundaries. The sample size needed for a significant difference is 1341 and 1968 for mortality at 24 h and at 30 days, respectively (Fig. 4).

## Discussion

Treatment of severely injured patients has seen in the last decades a major switch from a therapy based on large infusion of crystalloids, or synthetic colloids, to an early aggressive coagulative resuscitation. This is due to the evidence of an increase in mortality with larger volume of infusions [14] and the clarification of their role in worsening coagulation due to their dilutional effect, promotion of acidosis and reduction of core temperature due to the coolant effect of room temperature infusions. Civilian and military studies [15–17] showed a survival benefit when transfusion of blood product in fixed ratio were implemented in the first hour of treatment of severe trauma patients instead of large crystalloids volumes. The mounting evidence brought to the release of the ESA/ESICM guidelines [18] on the traumatic bleeding that advocated for early coagulative support, reduction of crystalloids and coagulative monitoring with viscoelastographic methods.

These important progresses are not completely reflected in a proportional reduction of deaths from traumatic haemorrhage in the first hours after arrival at the trauma centre. The logical consequence was to test if bringing an effective and time-sensitive treatment to the patients on the field of the accident (i.e. as early as possible) had any effect on mortality as long the ‘scooping time’ to the trauma center was preserved. This reflects the necessity to improve the pre-hospital management of the patients even from the ‘coagulative’ point of view [16, 17].

This meta-analysis summarizes the findings in a pristine, and under- challenged, field of the emergency medicine. Whether an infusion of plasma in the pre-hospital setting could reduce mortality and morbidity is a new question that arises from the positive data of several large retrospective studies. Moreover, it yields a sound pathophysiologic substrate as highlighted by several animal studies. The available evidence relies on two outstanding, large, low-biased, randomized clinical trials [9, 10]. To perform a meta-analysis of two studies can be considered not significant enough due to the paucity of trial. However, other meta-analyses in the literature have been done with two trials [19]. A systematic review with a comparison of the results between the two studies with a full list of differences and similarities can be enough, but it eventually leads to a win-win game. In fact, the meta-analysis is a dynamic process where the literature tries to summarize the existing evidences and explore the eventual necessity to proceed with other studies in the same fields or if the definitive possible answer has been reached. We undertook this meta-analysis for several reasons. The two studies had an almost identical experimental design but were performed in different settings. The results from the two studies were divergent. Pooling the data from the two studies could give a more general response to the question as they added patients and slightly different settings each to the other. It is like conducting the same study in two different levels of assistance setting. The incidence of events (i.e. mortality and morbidity) was different between the two studies. Moreover, we recognize that it will be hard to conduct other similar trials in the same topic; the present study aims to investigate also if it would be reasonable and useful or superfluous.

The two papers showed contrasting conclusion as the Moore et al. [9] paper showed no role in reducing the mortality while the Sperry et al. [10] resulted in a sharp 30-day mortality reduction, mainly due to a fall in mortality in the first hours of treatment. Of note, none of the studies reported any incidence of adverse events related to the transfusion of blood product on the field of the crush and failed to show an increase of transfusion-associated morbidities (i.e. acute lung injury and multi-organ failure). Moreover, pre-hospital plasma has no negative effects on multiple organ failure and acute lung injury. The rate of these two severe events is not influenced by plasma administration.

The pre-hospital resuscitative maneuvers could play a pivotal role in some aspects of the history of the patients as the coagulation derangements. Plasma administration seems to reduce the 24-h mortality without an impact on the long-term mortality and on the morbidity.

This result is downgraded by the paucity of randomized trials that could be inserted in the analysis. Moreover, the trial sequential analysis suggests that a larger number of patients would be needed to reliably confirm the effect of the pre-hospital plasma transfusion on mortality. Mortality neither at 24 h nor at 1 month was in the refusion or in the futility area, confirming that the results at present are valid but to be confirmed with further studies.

Generalizability of the results from this meta-analysis can be challenged in several ways. Plasma administration is an effective but time-sensitive treatment. Several mechanisms could explain the effect on mortality as reduction in bleeding or coagulopathy, a diminution of the inflammatory response or endothelial dysfunction of trauma, or both [9, 20, 21]. Some previously published trials demonstrated that when plasma is included in the resuscitation protocol inside the trauma center, it improves results in severely injured patients [22–24]. In a setting in which plasma would be given immediately after the time of injury, these results can potentially improve.

Present meta-analysis suggests that the survival benefit is mainly confined to the first 24 h and it seems to have no effect on the long-term mortality. Still, there is a trend toward a lower mortality at 30 days, and this data is outside the futility area of the trial sequence analysis. This fact suggests that pre-hospital plasma may increase the survival of patients with a share of them that will eventually survive long-term and another share that are unsalvageable.

The two studies considered in the meta-analysis present few differences that could at least partially account for the uncertainty of the results, even though they were very similar for inclusion and exclusion criteria. Transportation times were slightly different. Where transportation time is not as long as in the PAMPer study (median reported time 40–42 min, range 33–53) [10], the survival benefit could be significantly lessened or null. To note, the Moore et al. trial did not show a reduction in mortality and had a slightly shorter transport time (24–28 min, range 19–34). This could be one of the reasons why in the Moore et al. trial, patients received less prehospital crystalloids (500–900 mL, range 0–1500) than in the PAMPer study (median 250 mL) with both trials designed with crystalloids infusion based on haemodynamic need. Moreover, in the PAMPer study, initial Glasgow Coma Scale (GCS) was < 8 in 46% of the patients and the 51% of patients received intubation on the field; conversely, in the Moore et al. trial, the median GCS on the arrival was 14 in the two groups. A higher share of blunt trauma was reported in the Moore et al. paper, and mortality was markedly different with Sperry et al. reporting mortality almost double than Moore et al.

These facts could account for the large number of patients still needed to have reliable results as suggested by the Trial Sequential Analysis. Although sound according to the pre-experimental knowledge, the sample size calculation was far from the target and these new evidence must be taken into account in the development of new studies. The TSA is meant to overcome some critical points calculating a sample size and a futility/reliable evidence area that consider the different incidence of events in the different experimental settings. The different results between the standard and the TSA analysis point out the same problem that more studies with more patients are needed to explore this hypothesis, but the preliminary data are toward a possible benefit of the use of plasma in the pre-hospital setting.

## Conclusion

Pre-hospital plasma infusion seems to reduce 24-h mortality in haemorrhagic shock patients. It does not seem to influence 1-month mortality and acute lung injury and multi-organ failure rate.

## Abbreviations

CCTR: Cochrane Central Register of Controlled Trials
FFP: Fresh frozen plasma
ITT: Intention-to-treat
OR: Odds ratio
RCT: Randomized controlled trial
RR: Risk ratio
SBP: Systolic blood pressure
